# Efficacy of standard operating procedures for fall protection in hospitalized patients with schizophrenia

**DOI:** 10.1038/s41537-023-00396-3

**Published:** 2023-10-17

**Authors:** Hui Li, Caixing Liu, Zengyun Ge, Xishu Mu, Xuan Wang, Meihong Xiu, Xinfu Wang, Zezhi Li

**Affiliations:** 1 Hebei Province Veterans Hospital, Baoding, China; 2 Qingdao Mental Health Center, Qingdao, China; 3 Peking University HuiLongGuan Clinical Medical School, Beijing HuiLongGuan Hospital, Beijing, China; 4 Department of Nutritional and Metabolic Psychiatry, The Affiliated Brain Hospital of Guangzhou Medical University, Guangzhou, China; 5 Guangdong Engineering Technology Research Center for Translational Medicine of Mental Disorders, Guangzhou, China; 6 Key Laboratory of Neurogenetics and Channelopathies of Guangdong Province and the Ministry of Education of China, Guangzhou Medical University, Guangzhou, China

**Keywords:** Schizophrenia, Psychosis

## Abstract

Fall-related injury is the most common cause of functional disability and mortality in the older population. Falls in patients with schizophrenia are one of the major concerns in psychiatric hospitals. This study aimed to examine the impact of standardized operating procedures (SOP) on falls in veterans with schizophrenia. Veterans with schizophrenia were allocated to the control group (*n* = 345) and to the fall protection standardized operating procedures (FP-SOP) group (*n* = 342). Patients in the control group were given routine nursing for falls, and patients in the FP-SOP group were intervened with FP-SOP plus routine nursing. All patients were observed for one year. The study methods comply with the Strengthening the Reporting of Observational Studies in Epidemiology (STROBE) checklist. We found a fall rate of 1.5% in the FP-SOP group and 4.6% in the control group, with a significant difference in the fall rate between the two groups. In addition, the difference in patient satisfaction between the two groups was statistically significant. Our findings suggest that FP-SOP is an effective strategy for fall prevention in psychiatric hospitals.

## Introduction

Fall-related injuries are the leading cause of hospitalization, functional disability, and death among older adults^1–3^. Falls are also related to death from serious injury and increased emergency department visits and hospitalizations in patients with mental disorders^4,5^. A recent meta-analysis showed that older adults (≥60 years) with mental disorders have an annual falling incidence of 8.74% or a lifetime fall rate of 17.25%^6^.

Notably, a proportion of patients with mental disorders require long-term treatments with antipsychotics in psychiatric hospitals in closed settings^7^. In addition, patients are in a state of mental retardation, fatigue, and low motivation for self-care^1^. Long-term treatment with antipsychotics is more likely to cause side effects such as muscle relaxation and a greater risk of falling or falling from bed than in the general population^8^. Patients with SZ are more prone to suffer from osteoporosis, orthostatic hypotension, and frailty^9–12^. Therefore, reducing the incidence of falls in inpatients and ensuring patient safety is a compelling healthcare issue of psychiatric nursing management.

Over the past decade, studies on fall prevention and management have contributed to the development of fall guidelines^13–16^, as well as further evidence regarding the potential risks of falls in long-term care facilities^12,17^. Particularly, an 18-month observational cohort study in aged patients with acute psychosis reported that targeting patients over the age of 82 or diagnosed with dementia may reduce future falls, while targeting male patients with mental disorders and diagnosed with dementia may reduce recurrent falls on admission^18^. Moreover, effective dialogue between patients who report falls and clinical staff may support future remedial interventions and information to reduce the risk of falls^18^. Currently, in psychiatric units, studies on falls in patients with SZ have mainly focused on changes in inpatient facilities, health training and education, early intervention, and improvements in management. For example, Tao et al. showed that early nursing intervention after assessment significantly reduced the incidence of falls in China^19^. Chen et al. reported that a standard procedure provides predictive care for individuals at high risk of falls, which can significantly decrease the rate of falls^20^. However, it is not evident whether these fall prevention interventions can be easily implemented and adapted in psychiatric units. There is also a need to obtain the perspective of patients with SZ on these interventions.

It is known that there are many risk factors for falls in patients with SZ, therefore, fall prevention and intervention in psychiatric care settings should be evaluated from multiple perspectives. However, most previous research on falls in psychiatric patients has focused on changing environmental facilities, enhancing health education, early intervention, and strengthening management^21^. A comprehensive set of fall protection procedures for patients with SZ should be developed to be easily implemented in hospitals. The standardized operating procedure of fall protection (FP-SOP) was introduced into clinical nursing management of falls to analyze the risk factors of falls and develop an SOP for fall protection^20^. In addition, patient satisfaction goes to the acceptability of the intervention and understanding of patient acceptability would be important to other practitioners adopting the intervention.

In this study, we hypothesized that the FP-SOPs^20^ would be effective in preventing falls and improving patient satisfaction in hospitals. The purpose of this study was to investigate whether FP-SOP was effective for fall protection and achieved good patient satisfaction. To test this hypothesis, we examined three research questions in the present study. 1) Did participants in the study group fall fewer times than the comparison group? 2) What was the reason for falling in the two groups? And 3) Were the participants in the FP-SOP group satisfied with the intervention compared to the comparison group? Given the gender difference in the prevalence of falls among individuals^22^, we recruited only male patients.

## Methods

### Participants

SZ patients were recruited by advertisements in Hebei Province Veterans Hospital and all of the patients are hospitalized male patients with an extensive age range. Participants were consecutive patients who were admitted to a Veterans Affairs inpatient psychiatric unit between January 2018 and December 2019. The sample was representative of veterans with SZ on the unit and was all Chinese. 67.4% of the veterans were currently married, 19.9% were divorced, separated or widowed, and 12.7% were never married. Of all 687 patients, 190 were smokers (27.7%) and were non-smokers. The inclusion criteria were: (1) Diagnostic and Statistical Manual of Mental Disorders, fourth edition (DSM-IV) diagnosis of SZ and confirmed according to the Structured Clinical Interview for DSM-IV (SCID); (2) Han Chinese; (3) males; (4) age from 24 to 89 years; and (5) long-term hospitalization of 6 months or more. The exclusion criteria: (1) abuse or substance dependence except for tobacco; (2) self-injury or destructive and unprovoked violence or suicide in the past 6 months, as assessed by the Nurses’ Global Assessment of Suicide Risk (NGASR)^23^; (3) bedridden patients; (4) uncooperative patients within the last three days as assessed by the nurses; and (5) patients with consciousness disorders. The average age of all participants was 57.6 ± 9.9 years.

This study was conducted between January 2018 and December 2019. The study protocol was approved by the Institutional Review Boards (Ethic No.: IRBHPVH. 2018010). Each participant provided informed consent and signed a written informed consent form.

### Intervention

This is a quasi-experimental study using the rate of falls and patient satisfaction as the outcomes. All recruited inpatients were observed for one year in the current study. Control subjects (*n* = 345) received routine protective awareness training sessions, i.e., the nurses gave lectures on fall prevention knowledge every Tuesday afternoon in the ward activity room. In addition, they posted warning signs to prevent falls in the dining room and toilet. The average age of patients in the control group was 57.87 ± 9.71 years, with a range of 26–89 years. The duration of hospitalization was 49.54 ± 22.29, with a range of 2–76 months. There were 119 patients in the no risk group, 185 patients in the low-risk group, 35 patients in the medium-risk group and 6 patients in the high-risk group.

In addition to a regular educational session provided by the nurses, patients in the FP-SOP group (*n* = 342) also received an FP-SOP for fall prevention for 30 minutes every Tuesday afternoon in the ward activity room. The age of patients in the FP-SOP group ranged from 24 to 89 years, with an average of 57.37 ± 10.14 years. The duration of hospitalization was 55.56 ± 28.12 months, ranging from 6 to 87 months. There were 103 patients in the no-risk group, 192 patients in the low-risk group, 41 patients in the medium-risk group and 6 patients in the high-risk group. The FP-SOP group had a longer duration of hospitalization than the control group (*t* = 3.11, *p* = 0.002). There was no significant difference in age and risk level between the two groups (all *p* > 0.05).

In brief, the procedure of FP-SOP^20^ includes: in the first session, eleven nurses analyzed the potential risk factors associated with falls, and then they developed the manual of intervention guidelines for fall prevention according to the levels of fall risks. Sequent session: adopting different education sessions as patients’ age, educational levels and comprehension, teaching patients with a high level of literacy to develop fall prevention awareness through brochures and lectures, modifying the environment in the wards. Final session: changing patient behaviors. The nurses told the patients about prevention interventions based on each patient’s specific risks for possible falls, which convinced the patient of the fall prevention interventions and reduced potential fall risk behaviors. The prevention interventions were the same for all patients with the same level of fall risk. The setting for the FP-SOP group and the control group is the same ward.

### Assessments

The primary outcome measure was the fall incidence. Fall detection was based on a previous study^24^. We defined the fall incidence as the number of falls per the number of occupied bed days.

Risk factors for falls were assessed using the inpatient fall risk assessment form as the Hendrich II Fall Risk Model (HFRM II) fall-risk assessment scale^25^. It consists of 8 items and its total score is 16 points. The eight items are (1) Confusion, disorientation, impulsivity (4 points); (2) symptomatic depression (2 points); (3) altered elimination (1 point); (4) dizziness vertigo (1 point); (5) male gender (1 point); (6) any administered antiepileptics (2 points); (7) any administered benzodiazepines (1 point); Get up & Go test: (8) able to rise in a single movement-no loss of balance with steps (0 points); (9) pushes up, successful in one attempt (1 point); (10) multiple attempts, but successful (3 points); and (11) unable to rise without assistance during the test (4 points). There were 4 fall risk levels: no fall risk (0 points); low fall risk (1–2 points); medium fall risk (3-5 points); high fall risk (≥6 points). It has been translated into Chinese and validated the reliability and validity in elderly inpatients^26^. In addition, records were made of falls to clarify the reasons for falls that occurred during this study.

The second outcome measure was the patient satisfaction. The patient satisfaction questionnaire was assessed using a scale developed by the nursing department in our hospital (internal consistency Cronbach’s α = 0.851). It comprises 10 items that are used to calculate 3 index scores, including nurses’ service attitude, disease-related knowledge and fall-related knowledge. Each item is divided into very satisfied, satisfied and dissatisfied.

### Statistical analysis

**Fig. 1:**
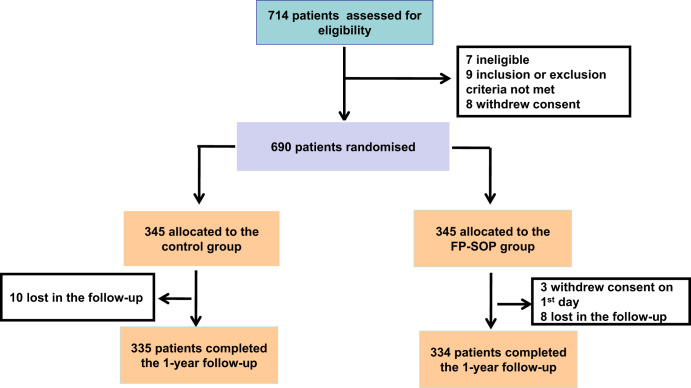
Study flow chart of this study. FP-SOP fall protection standardized operating procedures.

Data missing for patients who dropped out were imputed via the last observation carried forward (LOCF) method. All analyses to evaluate between-group differences were conducted for an intent to treat (ITT) analysis set that included the patients who received treatment. Three patients in the FO-SOP group were excluded since they withdrew their consent at the start of the study and did not receive treatment (Fig. 1). Demographic characteristics and falls in patients are presented as mean ± standard deviation for continuous variables and expressed as a number of patients along with percentages for categorical variables. The variables were tested for normal distribution in the control group and FP-SOP groups using the Shapiro-Wilk test. The demographic and clinical characteristics and long-term consequences of patients across the two groups are shown in 2 tables. To compare the age difference and clinical characteristics between the two groups, an analysis of variance (ANOVA) was performed for continuous variables and an *X*^2^ test for categorical variables^27,28^. The rates of falls and satisfaction were analyzed by using the *X*^2^ test and Fisher exact test.

The G*Power 3.1.9.2 software was performed to calculate the power^29^. PASW Statistics 20.0 software (SPSS Inc., Chicago, IL, USA) was performed in this study. All p values were 2-tailed at the significance level of <0.05.

## Results

### Comparison of the rate of falls between the two groups

The average age of patients who fell in the FP-SOP group was greater than in the control group (*t* = 2.03, *p* = 0.057) (Table 1). The fall rate was higher in the high-risk patients than in the medium-risk patients in both groups (*p* < 0.05, effect size = 0.33), with a power greater than 0.95.

**Table 1:** Clinical characteristics in the FP-SOP and control groups.

Item	Control group *n* = 345	FP-SOP group *n* = 342
Age (ys)	57.9 ± 9.7	57.4 ± 10.1
Duration of hospitalization (ms)	49.5 ± 22.3	55.6 ± 28.1
Onset age (ys)	24.1 ± 6.5	22.5 ± 3.0
Age at first hospitalization (ys)	26.3 ± 7.5	22.7 ± 3.2
Hospitalization times*	3.7 ± 2.8	4.8 ± 2.7
Duration of illness (ys)	24.9 ± 10.8	24.0 ± 10.0
*Marital status (n, %)*		
Married	224 (64.9)	239 (69.9)
Divorced	69 (20)	68 (19.9)
Single	52 (15.1)	35 (10.2)
Age (ys)^a^*	59.8 ± 11.4	66.2 ± 3.19
Smokers (*n*, %)	93 (27.0)	97 (28.4)
Education years (ys)	9.7 ± 2.6	9.4 ± 2.6
*Fall risk levels (n, %)*		
No fall risk	119 (34.5)	116 (33.9)
Low fall risk	185 (53.6)	184 (53.8)
Medium fall risk	35 (10.1)	35 (10.2)
High fall risk	6 (1.7)	7 (2.0)

^a^Age of the patients who fell in this study.

*ys* years, *ms* months.

^*^p < 0.05.

In the control group, there were 12 falls in the medium-risk patients, with a fall rate of 34.28%. For the high-risk patients in the control group, there were 3 falls with a fall rate of 50%. In the FP-SOP group, there were 4 cases of falls in the medium-risk patients, and the fall rate was 11.4%. There was 1 case of falls in high-risk patients, and the fall rate was 14.3%. In addition, we found that the rate of falls in the high and medium-risk patients in the FP-SOP group was significantly lower than that in the control group (5/342 vs16/345, *X*^*2*^ = 4.51, *p* = 0.034) (Table 2).

**Table 2:** The rate of falls for 1 year in the two groups with the Chi-square analysis.

	FP-SOP group Falls (*n*, %)	Control group Falls (*n,* %)	*F*(p)
Total sample	5/342 (1.5%)	16/345 (4.6%)	4.5 (0.03)
No fall risk	0/116	0/119	
Low fall risk	0/184	0/185	
Medium fall risk	4/35 (11.4%)	12/35 (34.3%)	3.3 (0.06)
High fall risk	1/7 (14.3%)	3/6 (50%)	1.0 (0.34)
*Fall sites*			
Toilets	–	4 (1.2%)	
Bed	3 (0.9%)	4 (1.2%)	
Restaurant and activity room	–	3 (0.9%)	
Ward, corridor	–	2 (0.6%)	
Outdoor	1 (0.3%)	2 (0.6%)	
Bathroom (n)	1 (0.3%)	1 (0.3%)	

### Reasons for falls among inpatients between the two groups

In the control group, there were 16 cases of falls. Among them, 6 falls were due to side effects induced by antipsychotic medication, 4 falls were due to psychiatric symptoms, 3 falls were due to mobility problems, 1 fall was due to a fall out of bed and 2 falls were due to environmental risk factors. In the FP-SOP group, there were 5 cases of falls, three of which were due to antipsychotic medication, 1 case due to psychiatric symptoms and 1 case due to mobility problems.

### Comparison of patient satisfaction between the two groups

Of patients in the control group, 345 patient satisfaction questionnaires were collected and 340 were considered as valid. The valid rate was 98.55%. Of patients in the FP-SOP group, 342 patient satisfaction questionnaires were collected and 336 were considered as valid. The valid rate was 98.24%. As shown in Table 2, the rate of patient satisfaction was significantly higher in the FP-SOP group than in the control group (*X*^*2*^ = 11.32, *p* = 0.01) (Table 3).

**Table 3:** The patient satisfaction for 1 year in the two groups with the Chi-square analysis.

Group	*n*	Very satisfied	Satisfied	Dissatisfied	*X*^2^ (*p*)
Control	340	51 (15%)	251 (73.8%)	38 (11.2%)	11.3 (0.01)
FP-SOP	336	102 (30.4%)	222 (66.1%)	12 (3.6%)	

## Discussion

We found that FP-SOP significantly decreased the rate of falls among veterans with SZ compared to the control group. In addition, the rate of patient satisfaction was higher in the FP-SOP group than in the control group.

### Efficacy of FP-SOP on fall prevention

Falls can cause varying degrees of injuries to patients with SZ. It not only increases patients’ pain and prolongs the duration of hospitalization, but also imposes a financial burden on families^30^. Falls and fall-related injuries have been recognized as an increasing health problem^31^. Therefore, preventive interventions for patients are needed, especially for those at moderate or high-risk levels. Studies have shown that all older adults should be advised on falls prevention to improve quality of life^32–35^. Our findings showed that the higher the risk assessment level, the greater the likelihood of falls, indicating the higher reliability of the new version of the assessment forms. We generated FP-SOPs based on the risk levels assessed by the assessment form, minimized confounding factors and monitored the implementation process to ensure FP-SOPs were implemented.

It is worth noting that the patients in our study were older, long-term hospitalized patients with SZ. Although the number of hospital falls has decreased in the last few years, it is still a serious problem^36^. Previous studies reported that psychiatric hospitals used a variety of “guidelines” for fall prevention that include (1) identifying patients at high risk for falls, and (2) using clinical judgment to determine which of multiple fall prevention approaches to reduce fall risk^37–40^. However, the lack of clarity in prevention guidelines may increase confusion about the “right way” to prevent falls, increase the cognitive burden of patient care, be labor-intensive and time-consuming, and may increase the risk of falls^30,41^. This study is the first to implement the FP-SOP intervention in long-term hospitalized veterans with SZ. We found that implementing the FP-SOP intervention for patients at medium or high risk did reduce the rate of falls. FP-SOP is a combination of prevention of multiple fall-related risk factors by increasing knowledge of falls and preventing risk behaviors that predispose patients to falls. We found 16 falls in the control group compared to 5 falls in the FP-SOP group. The incidence of falls among long-term veterans decreased significantly from 4.64% to 1.46%, which is consistent with several previous studies^42^. Although there was no difference in risk levels between the FP-SOP and control groups, FP-SOP significantly reduced the rates of falls, especially among patients at medium to high risk in the FP-SOP group. This study suggests that FP-SOP is effective in fall prevention in long-term hospitalized patients with SZ and can be used in clinical practice.

### Patient satisfaction with FP-SOP

Patient satisfaction is a critical indicator of high-quality nursing care. Implementation of new fall prevention strategies can preserve patient health. We speculated that there should be a relationship between FP-SOP implementation and patient satisfaction. Patient satisfaction may be one of the key indicators of the quality of FP-SOP implementation. Specifically, after the implementation of FP-SOP, we found a statistically significant increase in patient satisfaction, from 88.83% to 96.43%. The generation and implementation of FP-SOP is an ongoing process, and the charge nurse plays a pivotal role in the implementation process. Therefore, nurse training becomes important for the implementation of FP-SOP to achieve goals and quality control. Previous studies have shown that patients will not be able to comply with the nurse’s instructions if they do not completely understand them. Nurses may contribute to the occurrence of errors that affect patient satisfaction, especially in the longest serve time (24 consecutive hours) and most frequent nurse-patient interactions for various nursing actions.

### Strengths and limitations

This is a very important finding because we validated the effectiveness of standard operating procedures for fall prevention in patients with SZ. To our best knowledge, this is the first study to use FP-SOP to prevent falls in high-risk patients with SZ. However, there were several limitations of this study. First, the sample in this study was limited to men and cannot be generalized to women. Further study should be conducted to assess whether the efficacy of FP-SOP in women is similar to that in men. Second, although the sample size was large, the rate of falls among patients with SZ was low. Therefore, we were unable to compare the rate of falls in the two groups at different time points. Third, we did not collect the type and dose of antipsychotics taken by the patients, but an early study by Chang et al. has suggested that a fall prevention intervention should be considered^43^. Forth, the fall prevention reporting was conducted at a psychiatric hospital for only 12 months. Fifth, a potential selection bias cannot be ruled out since participants tend to be more compliant than those who refuse to participate in the study. In addition, there may be a performance bias since the patients were not blind in the current study. We also cannot exclude the potential measurement bias of the assessment of risk factors for falls and falls. Sixth, the satisfaction of FP-SOP was not targeted on the users, i.e., the nurses in this study. Nurse compliance in the implementation of FP-SOP is very influential in preventing patients from falling, because the patient’s family may not be directly involved in the implementation of the fall prevention interventions.

## Conclusions

Physical health care in people with SZ still needs attention, especially for those who are hospitalized for longer periods. Our findings provide a new intervention for patients with SZ in psychiatric hospitals. Future studies are necessary to verify whether FP-SOP targeting multilevel fall risk factors can prevent falls in patients with other psychiatric disorders, such as major depressive disorders.

### Nursing implications

Falls are a significant problem for the healthcare system. This is an ambitious attempt to develop a standard FP-SOP for fall prevention and management in psychiatric hospitals. Patients in psychiatric units have unique risk factors for falls. Most previous studies about in-hospital fall prevention have focused on general acute care rather than on psychiatric units. The FP-SOP procedure has focused on patients with mental disorders in psychiatric units. Our studies demonstrate the importance of implementing FP-SOP for fall prevention in nursing care in psychiatric units. It is not only effective in preventing falls, but also in increasing patient satisfaction.

Patients with SZ on long-term antipsychotic medication are more likely to fall in the wards due to lack of physical exercise and changes in medication regimens or the inclusion of multiple medications. The vast majority of patients are unaware of fall prevention procedures, even if they have experienced one or more fall events or have received multiple verbal fall prevention education. Therefore, we believe the FP-SOP intervention for fall prevention can help reduce fear and insecurity among SZ patients in psychiatric units. Because our FP-SOP was generated by experienced nurses in psychiatric units, it is pragmatic and adaptable for long-term inpatients in different scenarios. Our FP-SOP for fall prevention better balances the promotion of independent self-care with the need to provide more hands-on assistance to prevent falls.

## Data Availability

The datasets generated during and/or analyzed during this study are available from the corresponding author upon reasonable request.
